# First report of *Aphrialatifrons* (Diptera, Tachinidae, Leskiini) in the Canary Islands

**DOI:** 10.3897/BDJ.11.e109690

**Published:** 2023-09-21

**Authors:** Daniel Suárez, David Lugo, Mónica Pérez-Gil, Gustavo Peña, Carlos Ruiz

**Affiliations:** 1 Island Ecology and Evolution Research Group, Instituto de Productos Naturales y Agrobiología (IPNA-CSIC), La Laguna (Tenerife), Spain Island Ecology and Evolution Research Group, Instituto de Productos Naturales y Agrobiología (IPNA-CSIC) La Laguna (Tenerife) Spain; 2 Departamento de Biología Animal, Edafología y Geología, Facultad de Ciencias, Universidad de La Laguna, La Laguna (Tenerife), Spain Departamento de Biología Animal, Edafología y Geología, Facultad de Ciencias, Universidad de La Laguna La Laguna (Tenerife) Spain; 3 Cetaceans and Marine Research Institute of the Canary Islands, San Bartolomé (Lanzarote), Spain Cetaceans and Marine Research Institute of the Canary Islands San Bartolomé (Lanzarote) Spain

**Keywords:** new record, distribution, parasitoid, Macaronesia

## Abstract

**Background:**

The Canary Islands are an archipelago of volcanic origin, located off north-west Africa comprising eight islands. Fuerteventura and Lanzarote are the oldest (20 and 15 millon years old, respectively) and the easternmost islands. The order Diptera is one of the most relevant taxa in the Canary Islands as they constitute the second highest species richness. Within this order, the family Tachinidae is especially interesting as all species are endoparasitoids of arthropods and most species play a key role as pollinators. In the Canary Islands, the family comprises 52 species, with Fuerteventura and Lanzarote harbouring up to 20 species each.

**New information:**

*Aphrialatifrons*, a Palaearctic tachinid fly, is reported for the first time from the Canary Islands, where it was found on Fuerteventura and Lanzarote. Morphological examination was carried out and the first known barcode of the species is presented. Its potential distribution and source of origin are discussed.

## Introduction

The Canary Islands are an archipelago of volcanic origin, located off north-west Africa comprising eight islands. Fuerteventura and Lanzarote are the oldest (20 and 15 million years old, respectively) and the easternmost islands, being only 96 km off the African coast (Troll and Carracedo 2016). Due to a comparatively low altitude, the humidity of the tradewinds is not retained, which results in little precipitation and desert-like conditions. Together with erosion and aridification, Fuerteventura and Lanzarote exhibit a reduced habitat diversity compared to younger central and western islands (Macías-Hernandez et al. 2016). The order Diptera is one of the most relevant taxa in the Canary Islands as it constitutes the highest species richness (being only superseded by the hyperdiverse Coleoptera), with a total number of 1,167 species, almost a third of these endemic to the Islands (Gobierno de Canarias 2023). The family Tachinidae is especially interesting as all species are endoparasitoids of arthropods (Stireman et al. 2019) and most species play a key role as pollinators (Tooker et al. 2006). In the Canary Islands, the family comprises 52 species, with Fuerteventura and Lanzarote harbouring up to 20 species each (Gobierno de Canarias 2023). There is a strong separation in the tachinid species assemblage of Fuerteventura and Lanzarote compared to the remaining islands, with a high percentage of exclusive species, most of them being Mediterranean and distributed in coastal areas (Suárez et al. 2020). This pattern could be driven by the similarity with the neighbouring arid part of the African continent (Báez et al. 1986). In order to contribute to the knowledge of the Tachinidae fauna of the Canary Islands, we provide a first report of the genus *Aphria* Robineau-Desvoidy, 1830, based on specimens observed and collected on Fuerteventura and Lanzarote.

## Materials and methods

A single specimen was hand-collected, pinned and examined under a Zeiss Stemi 2000 stereomicroscope. The specimen was identified using a dichotomous key of Western Palaearctic-inhabiting species of the genus *Aphria* (Cerretti 2010), as well as with original descriptions including Eastern Palaearctic and Nearctic species (Townsend 1891, Townsend 1908, Villeneuve 1908, Mesnil 1963, Richter 1978, Herting 1984). DNA was extracted from one leg using NucleoSpin Macherey-Nagel DNA extraction kit. The 5’ region (658 bp) of the mtDNA COI gene was amplified using LCO1490 and HCO2980 primers (Folmer et al. 1994). PCR reaction conditions were as follows: initial denaturation at 95°C for 2 min, followed by 35 cycles of 94°C for 45 s, 40°C for 35 s and 72°C for 45 s and a final extension of 72°C for 7 min. Two μl of diluted (1/10) DNA extract was amplified with 23 μl of PCR mix (for a total volume of 25 μl), comprised of 8.5 μl of water, 1 μl of each primer (10 μM) and 12.5 μl of REDTaq ReadyMix PCR Reaction Mix. PCR products were sequenced using the Sanger DNA sequencing service of Macrogen. Sequences were then edited in Geneious 2021.1.1 and compared to the database of BOLD. A dataset was assembled including our sequenced barcode, a set of available barcode sequences within the genus *Aphria* and a closely-related species (outgroup). A Maximum Likelihood phylogenetic tree was built using RAxML 8.2.11 (Stamatakis 2014). The specimen was deposited in the collection of the Department of Animal Biology of the University of La Laguna (DZUL). Additionally, more specimens were photographed in their natural habitat using an Olympus E-M1MarkIII camera. Plants being pollinated were annotated, with botanical taxonomy following ‘Plants of the World Online’.

## Taxon treatments

### Aphria latifrons

Villeneuve, 1908

44D2B166-A774-5637-97F1-8D030F4030D7

#### Materials

- **Type status:** Other material. **Occurrence:** catalogNumber: DZUL-B728; recordedBy: Mónica Pérez-Gil; individualCount: 1; lifeStage: adult; associatedSequences: BOLD: INSCI001-23; occurrenceID: 3E2BDDDB-4E03-529D-8A7F-5627A1A618CF; **Taxon:** taxonID: https://www.gbif.org/es/species/7793046; scientificName: *Aphrialatifrons* Villeneuve, 1908; order: Diptera; family: Tachinidae; genus: Aphria; **Location:** island: Lanzarote; country: Spain; stateProvince: Las Palmas; municipality: Teguise; locality: Caleta de Famara; decimalLatitude: 29.122276; decimalLongitude: -13.57246; georeferenceProtocol: GPS; **Identification:** identifiedBy: Daniel Suárez; dateIdentified: 2023; **Event:** eventDate: 11/09/2022; eventRemarks: On flowers of *Caroxylonvermiculatum* (Amaranthaceae); **Record Level:** institutionCode: DZUL; basisOfRecord: PreservedSpecimen
- **Type status:** Other material. **Occurrence:** recordedBy: Mónica Pérez-Gil; individualCount: 1; lifeStage: adult; occurrenceID: 47EB19C5-741C-5761-A073-94F60352C6E4; **Taxon:** taxonID: https://www.gbif.org/es/species/7793046; scientificName: *Aphrialatifrons* Villeneuve, 1908; order: Diptera; family: Tachinidae; genus: Aphria; **Location:** island: Lanzarote; country: Spain; stateProvince: Las Palmas; municipality: Yaiza; locality: Salinas del Janubio; decimalLatitude: 28.932979; decimalLongitude: -13.826846; georeferenceProtocol: GPS; **Identification:** identifiedBy: Daniel Suárez; dateIdentified: 2023; **Event:** eventDate: 01/12/2023; eventRemarks: On flowers of *Zygophyllumfontanesii* (Zygophyllaceae); **Record Level:** basisOfRecord: HumanObservation
- **Type status:** Other material. **Occurrence:** recordedBy: Mónica Pérez-Gil; individualCount: 1; lifeStage: adult; occurrenceID: 3F06C1F6-583B-51C5-86BC-CEF3E858119D; **Taxon:** taxonID: https://www.gbif.org/es/species/7793046; scientificName: *Aphrialatifrons* Villeneuve, 1908; order: Diptera; family: Tachinidae; genus: Aphria; **Location:** island: Lanzarote; country: Spain; stateProvince: Las Palmas; municipality: Haría; locality: Órzola; decimalLatitude: 29.225886; decimalLongitude: -13.455319; georeferenceProtocol: GPS; **Identification:** identifiedBy: Daniel Suárez; dateIdentified: 2023; **Event:** eventDate: 01/11/2023; eventRemarks: On flowers of Senecioleucanthemifoliusvar.falcifolius (Asteraceae); **Record Level:** basisOfRecord: HumanObservation
- **Type status:** Other material. **Occurrence:** recordedBy: Mónica Pérez-Gil; individualCount: 5; lifeStage: adult; occurrenceID: 85775AB7-095D-5484-8C30-36BFA0308268; **Taxon:** taxonID: https://www.gbif.org/es/species/7793046; scientificName: *Aphrialatifrons* Villeneuve, 1908; order: Diptera; family: Tachinidae; genus: Aphria; **Location:** island: Lanzarote; country: Spain; stateProvince: Las Palmas; municipality: Haría; locality: La Cantería; decimalLatitude: 29.224659; decimalLongitude: -13.460386; georeferenceProtocol: GPS; **Identification:** identifiedBy: Daniel Suárez; dateIdentified: 2023; **Event:** eventDate: 01/11/2023; eventRemarks: On flowers of Senecioleucanthemifoliusvar.falcifolius (Asteraceae) and *Zygophyllumfontanesii* (Zygophyllacceae); **Record Level:** basisOfRecord: HumanObservation
- **Type status:** Other material. **Occurrence:** recordedBy: Mónica Pérez-Gil; individualCount: 1; lifeStage: adult; occurrenceID: C8FD5643-DA67-5C8C-A38A-2114FAB2A872; **Taxon:** taxonID: https://www.gbif.org/es/species/7793046; scientificName: *Aphrialatifrons* Villeneuve, 1908; order: Diptera; family: Tachinidae; genus: Aphria; **Location:** island: Lanzarote; country: Spain; stateProvince: Las Palmas; municipality: Teguise; locality: Las Laderas; decimalLatitude: 29.09195; decimalLongitude: -13.556546; georeferenceProtocol: GPS; **Identification:** identifiedBy: Daniel Suárez; dateIdentified: 2023; **Event:** eventDate: 02/28/2023; eventRemarks: On flowers of *Asteriscusintermedius* (Asteraceae); **Record Level:** basisOfRecord: HumanObservation
- **Type status:** Other material. **Occurrence:** recordedBy: Mónica Pérez-Gil; individualCount: 1; lifeStage: adult; occurrenceID: 67A6F2D4-6492-50A9-BFF7-73B32A6517E2; **Taxon:** taxonID: https://www.gbif.org/es/species/7793046; scientificName: *Aphrialatifrons* Villeneuve, 1908; order: Diptera; family: Tachinidae; genus: Aphria; **Location:** island: Lanzarote; country: Spain; stateProvince: Las Palmas; municipality: Teguise; locality: Barranco de la Espoleta; decimalLatitude: 29.050151; decimalLongitude: -13.4667581; georeferenceProtocol: GPS; **Identification:** identifiedBy: Daniel Suárez; dateIdentified: 2023; **Event:** eventDate: 09/08/2023; eventRemarks: On flowers of *Caroxylonvermiculatum* (Amaranthaceae); **Record Level:** basisOfRecord: HumanObservation
- **Type status:** Other material. **Occurrence:** recordedBy: Mónica Pérez-Gil; individualCount: 1; lifeStage: adult; occurrenceID: 21D29825-2ECA-52DF-AAC8-F7C2318BA5D1; **Taxon:** taxonID: https://www.gbif.org/es/species/7793046; scientificName: *Aphrialatifrons* Villeneuve, 1908; order: Diptera; family: Tachinidae; genus: Aphria; **Location:** island: Lanzarote; country: Spain; stateProvince: Las Palmas; municipality: Haría; locality: Playa de Punta Prieta; decimalLatitude: 29.199928; decimalLongitude: -13.421317; georeferenceProtocol: GPS; **Identification:** identifiedBy: Daniel Suárez; dateIdentified: 2023; **Event:** eventDate: 11/30/2021; eventRemarks: On flowers of *Traganummoquinii* (Chenopodiaceae); **Record Level:** basisOfRecord: HumanObservation
- **Type status:** Other material. **Occurrence:** recordedBy: Mónica Pérez-Gil; individualCount: 1; lifeStage: adult; occurrenceID: 9CBF5253-3D4B-52DA-9336-B0C3169F1A3D; **Taxon:** taxonID: https://www.gbif.org/es/species/7793046; scientificName: *Aphrialatifrons* Villeneuve, 1908; order: Diptera; family: Tachinidae; genus: Aphria; **Location:** island: Lanzarote; country: Spain; stateProvince: Las Palmas; municipality: Teguise; locality: Caleta de Caballo; decimalLatitude: 29.119734; decimalLongitude: -13.641454; georeferenceProtocol: GPS; **Identification:** identifiedBy: Daniel Suárez; dateIdentified: 2023; **Event:** eventDate: 11/12/2022; eventRemarks: On flowers of Caroxylonvermiculatum (Amaranthaceae),; **Record Level:** basisOfRecord: HumanObservation
- **Type status:** Other material. **Occurrence:** recordedBy: Johan Verstraeten; individualCount: 1; lifeStage: adult; occurrenceID: 69FE5DFA-0D8B-5399-9841-19700C57AB39; **Taxon:** taxonID: https://www.gbif.org/es/species/7793046; scientificName: *Aphrialatifrons* Villeneuve, 1908; order: Diptera; family: Tachinidae; genus: Aphria; **Location:** island: Fuerteventura; country: Spain; stateProvince: Las Palmas; municipality: La Oliva; locality: Caldereta; decimalLatitude: 28.587634; decimalLongitude: -13.875284; georeferenceProtocol: GPS; **Identification:** identifiedBy: Daniel Suárez; dateIdentified: 2023; **Event:** eventDate: 02/23/2023; eventRemarks: On flowers of *Glebioniscoronaria* (Asteraceae); **Record Level:** basisOfRecord: HumanObservation

#### Diagnosis

**Figure 1.**
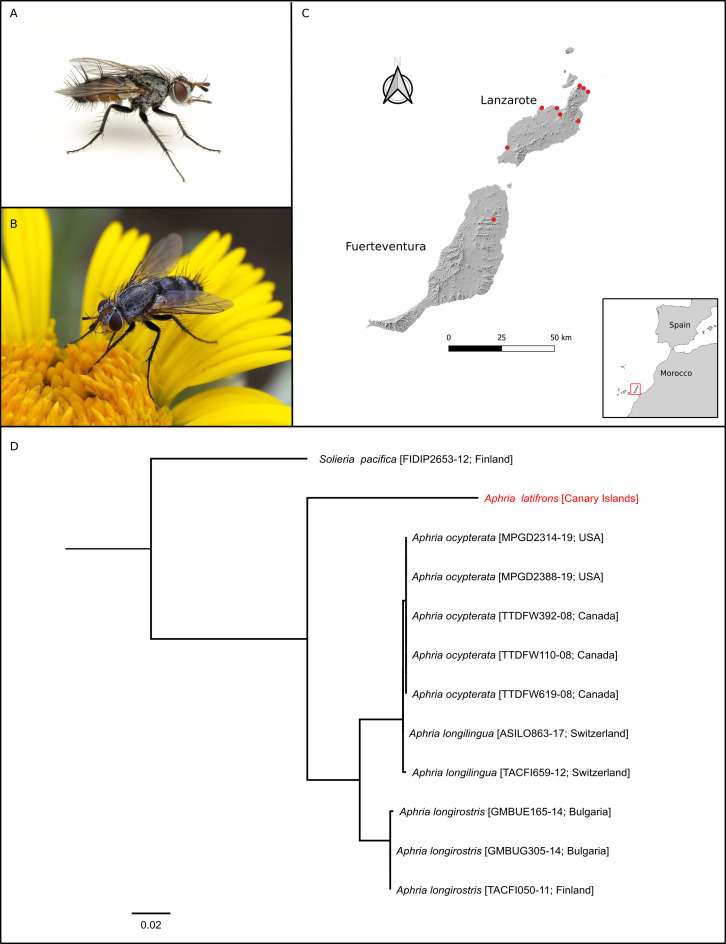
Habitus, distribution and genetic data for *Aphrialatifrons*. **A** Lateral view of living specimen of *Aphrialatifrons* (photo: M. Pérez-Gil); **B** Lateral view of living specimen of *Aphrialatifrons* (photo: M. Pérez-Gil); **C** Distribution maps of the islands of Fuerteventura and Lanzarote showing the observed distribution of *Aphrialatifrons* (red dots). The location of Fuerteventura and Lanzarote within the Canary Islands is marked with a red square in the inset; **D** Maximum-Likelihood tree of the genus *Aphria*. Countries are indicated after BOLD accession numbers. Red: specimen sequenced in this study.

Specimens were identified as *Aphrialatifrons* for having the following unique combination: yellow tegula, R4+5 bristles not reaching the intersection with R-M vein, CS4 shorter than CS6 (Fig. 1A and B).

#### Distribution

Tunisia, France, Italy, Spain, Switzerland, Russia, Transcaucasia, Kazakhstan and Mongolia (Villeneuve 1907, Herting 1984, Tschorsnig 2001). Distribution within Fuerteventura and Lanzarote is presented in Fig. 1C.

#### Habitat

The localities of La Cantería, Órzola and Punta Prieta are composed by a halophilic and poorly nitrophilous vegetation, physiognomically characterised by the presence of *Suaedavera* forming thickets with *Frankeniacapitata* and *Zygophyllumfontanesii*. In Salinas de Janubio, there is present a chamaephytic community, growing in a highly saline disturbed littoral, characterised by two common species of halophilic environments, *Zygophyllumfontanesii* and *Suaedavermiculata*. Las Laderas has a chamaephytic dwarf community formed by stunted chamaephytes growing on exposed windy and strongly grazed soils where *Helianthemumcanariense* and *Spergulariafimbriata* are dominant. The remaining localities (Caldereta, Caleta de Famara and Barranco de La Espoleta) are composed by nitrophilous synanthropic shrubs dominated by *Caroxylonvermiculatum*, *Suaedavermiculata* and *Bassiatomentosa* (del Arco Aguilar and Rodríguez Delgado 2018).

#### Genetic data

A 658-bp fragment was successfully amplified (BOLD accession code: INCSI001-23). The specimen shows an 8.66% of divergence (uncorrected p-distance) to specimens of *Aphriaocypterata* and *Aphrialongilingua*, as well an 8.12-8.89% of divergence to specimens of *Aphrialongirostris* (Fig. 1D).

#### Host

Hosts unknown. Probably Pyralidae, based on hosts of other species (Tschorsnig 2017).

## Discussion

Within the genus *Aphria*, *Aphriagracilis* Mesnil, 1963, *Aphrialongirostris* (Meigen, 1824) *Aphrialongilingua* Villeneuve, 1907 and *Aphriapotans* (Wiedemann, 1830) have a black tegula (Mesnil 1963, Cerretti 2010, Canadian National Collection 2023b). The Neartic species *Aphriageorgiana* Townsend, 1908 and *Aphriamiranda* Townsend, 1891 have a white tegula (Townsend 1891, Townsend 1908). Amongst the yellow-tegulae species, *A.xiphias* Pandellé, 1896 and *A.rubida* Mesnil, 1973 can be separated from *A.latifrons* by having R4+5 bristles that reach and exceed the intersection with the R-M vein (Cerretti 2010, Canadian National Collection 2023a). *A.miranda* Richter, 1978 can be separated from *A.latifrons* by having only two or three setae at the base of R4+5 (Richter 1978). The phylogenetic tree, based on a mitochondrial gene, placed *A.latifrons* as a basal clade compared to the remaining species (Fig. 1D), with a higher interspecific divergence (ca. 8%) compared to the one existing between the *ocypterata*-*longilingua*-*longirostris* clade (< 1% between *A.ocypterata* and *A.longilingua*, ca. 4% between *A.longirostris* and either *A.ocypterata* or *A.longilingua*). As robust inferences cannot yet be made due to an underrepresentation on public databases (only three species with sequences from a total of 10 valid species), further analyses including material from the remaining species will allow for a better understanding of the evolution of the genus.

This record of *Aphrialatifrons* on Fuerteventura and Lanzarote is not only the first report of the genus for the Canary Islands, but also for any of the four Macaronesian archipelagos (Arechavaleta et al. 2005, Borges et al. 2008, Borges et al. 2010, Gobierno de Canarias 2023). Due to its Palaearctic distribution, there is no evidence for considering the recent discovery of this species as an introduction. Instead, a recent natural dispersion may have occurred. Due to its known disjunct distribution, it is probable that this species is more widespread throughout the western Palaearctic and the geographic distance may be shorter than the current known distribution [ca. 1100 km from Andalusia (Spain) and ca. 2000 km from Tunisia]. Although it has not been reported for Morocco (Kettani et al. 2022), their close distance to Tunisia and the fact that the first report for a species of *Aphria* was relatively recent (Ebejer et al. 2019), may be an indication of underestimated diversity. If the species is found in Morocco, which is relatively close to Fuerteventura (96 km), a natural colonisation from North Africa may be thus more plausible. Specimens have been found in the northern and south-western parts of Lanzarote, 50 km distant from each other, both in coastal and interior areas, suggesting that this species could be more widespread along the Island. The specimen from Fuerteventura is 40 km distant from the southernmost recorded point in Lanzarote. The vegetation units where specimens were found are characteristic for most areas of the southern area of Lanzarote, as well as coastal areas from eastern and central areas and practically throughout all the Island of Fuerteventura. More sampling effort might uncover additional unknown populations of the species.

The biology of this species is not well studied. A 5-year-research carried out at the South Tyrol (Italy) revealed that adults were active from late-spring to early-autumn (Ziegler and Tschorsnig 2016). However, in Lanzarote, individuals were found mainly in winter, with most specimens being observed in January. This difference could be driven by the flowering phenology of Lanzarote, being closely related to rainfall episodes occurring in late-winter. Regarding floral resources, in the South Tyrol, it has been observed feeding on *Senecioinaequidens* (Asteraceae) and *Thymuspraecox* (Lamiaceae). In Fuerteventura and Lanzarote, it was observed feeding on six different plant species from the families Asteraceae, Amaranthaceae, Chenopodiaceae and Zygophyllaceae. To date, the hosts for *A.latifrons* are unknown. Other species of the genus *Aphria* are known to occur as parasites in moths of the genus *Sciota* Hulst, 1888 (Tschorsnig 2017). In the Canary Islands, the native non-endemic species *Neurotomiacoenulentella* (Zeller, 1846) [=*Sciotacoenunlentella* (Zeller, 1846)] inhabits only the Island of Fuerteventura (Arenberger 1999), with no further additional species of the genus being recorded. Further studies are needed to determine which is the host of *A.latifrons* on the Canary's Archipelago.

## Supplementary Material

XML Treatment for Aphria latifrons
